# Infective Endocarditis in Patients with Mitral Annular Calcification: Clinical and Echocardiographic Presentation, Management, and Outcomes

**DOI:** 10.1016/j.cjco.2025.10.020

**Published:** 2025-11-17

**Authors:** Gaspard Suc, Lawrence Lau, Serah Seo, Francois Auclair, Vicente Corrales-Medina, Vincent Chan, Ian G. Burwash, Roja Gauda, Fraser Rubens, Kwan L. Chan, David Messika-Zeitoun

**Affiliations:** a Division of Cardiology, University of Ottawa Heart Institute, Ottawa, Ontario, Canada; b Division of Infectious Diseases, Ottawa Civic Hospital, Ottawa, Ontario, Canada; c Division of Cardiac Surgery, University of Ottawa Heart Institute, Ottawa, Ontario, Canada; d School of Epidemiology and Public Health, Faculty of Medicine, University of Ottawa, Ottawa, Ontario, Canada

**Keywords:** infective endocarditis, echocardiography, mitral annular calcification

## Abstract

**Background:**

Mitral annular calcification (MAC) poses unique challenges in infective endocarditis (IE). This study aimed to characterize echocardiographic features of IE involving MAC and assess its management and outcomes.

**Methods:**

We reviewed cases discussed during our IE multidisciplinary meetings, 2021-2024. Clinical data, imaging findings, and outcomes were collected through chart review.

**Results:**

Among 741 patients evaluated, 51 patients (7%; aged 73 ± 11 years; 45% female) had possible or definite IE and moderate-severe MAC. IE involved the mitral valve in 24 patients (47%). Mitral IE was more frequent in women (63% vs 30%, *P* = 0.02) and was less commonly associated with prior aortic valve replacement (17% vs 59%, *P* < 0.01). *Staphylococcus aureus* was the most common pathogen, with no difference between groups (38% vs 30% in the mitral vs nonmitral IE groups, respectively, *P* = 0.55). Patients with mitral IE often presented with large vegetations (median 13 mm), frequent valvular or perivalvular complications (moderate or greater mitral regurgitation in 54%, perforation in 33%, and perivalvular abscess in 13%). A succulent aspect of the vegetation mass with abnormal MAC mobility, described as "rocking," was observed in 7 patients (29%). Surgical indications were found in 63% of patients, but only 40% underwent surgery due to high perceived risk. The in-hospital mortality incidence was not different between the mitral and nonmitral IE groups (29% vs 15%, *P* = 0.32).

**Conclusions:**

In patients with IE and MAC, the mitral valve was often primarily affected, showing large, mobile vegetations with a “rocking” motion. Despite frequent surgical indications, few underwent surgery, highlighting the need for improved management in this high-risk group.

## Résumé

**Contexte:**

Les calcifications de l'anneau mitral (MAC) posent des défis spécifiques en cas d'endocardite infectieuse (EI). Cette étude visait à préciser les caractéristiques échocardiographiques de l'EI associée à une MAC et à évaluer la prise en charge et les devenir des patients.

**Méthodologie:**

Nous avons examiné les cas discutés lors de nos réunions multidisciplinaires dédiées à l'EI entre 2021 et 2024. Les données, les résultats d'imagerie et les résultats cliniques ont été recueillis à partir de l'examen des dossiers.

**Résultats:**

Parmi les 741 patients évalués, 51 patients (7 %) (73 ± 11 ans, 45 % de femmes) présentaient une EI possible ou certaine et une MAC modérée/sévère. L'EI touchait la valve mitrale chez 24 patients (47 %). L'EI mitrale était plus fréquente chez les femmes (63 % contre 30 %, *p* = 0,02) et moins souvent associée à un remplacement valvulaire aortique antérieur (17 % contre 59 %, *p* < 0,01). *Staphylococcus aureus* était l'agent pathogène le plus fréquent, sans différence entre les groupes (38 % vs 30 % dans l'EI mitrale et non mitrale respectivement, *p* = 0,55). Les patients atteints d'EI mitrale présentaient souvent de larges végétations (médiane 13 mm), des complications valvulaires ou périvalvulaires fréquentes (régurgitation mitrale modérée ou supérieure dans 54 % des cas, perforation dans 33 % des cas et abcès périvalvulaire dans 13 % des cas). Un aspect succulent de la végétation associe à une mobilité anormale de la MAC, décrite comme un « mouvement de bascule », était observé chez 7 patients (29 %). Une indication chirurgicale théorique était présente chez 63 % des patients, mais seuls 40 % ont bénéficié d'une intervention chirurgicale en raison d’un risque perçu élevé. La mortalité hospitalière n'était pas différente entre les groupes EI mitrale et non mitrale (29 % contre 15 %, *p* = 0,32).

**Conclusion:**

Chez les patients atteints d'EI et de MAC, la valve mitrale était souvent principalement affectée, présentant des végétations larges et mobiles avec un mouvement de bascule. Malgré des indications chirurgicales fréquentes, peu ont été opérés, ce qui souligne la nécessité d'améliorer la prise en charge de ce groupe à haut risque.

Mitral annular calcification (MAC) is a common chronic degenerative condition that affects the fibrous annulus of the mitral valve,^1^ and its prevalence is increasing significantly as the population ages.^2^ MAC is linked to atherosclerosis and is associated with heightened risks of mortality, myocardial infarction, and stroke.^3^ In advanced cases, calcification can extend into the mitral leaflets, leading to calcific mitral valve disease.^4^, ^5^, ^6^ This advanced form of MAC can cause significant hemodynamic consequences, mitral stenosis, mitral regurgitation (MR), or a combination of both. Notably, calcific mitral valve disease has become the leading cause of mitral stenosis in Western countries.^7^

MAC presents significant surgical challenges, due to the advanced age and associated comorbidities of affected patients and the increased risk of major intraoperative and postoperative complications related to the extent of calcification, such as atrioventricular disruption and paravalvular regurgitation.^1^^,^^8^ Because of these challenges, MAC commonly is referred to as the “bar of death.”^9^ Although transcatheter therapies have been explored, they remain feasible in only a limited subset of patients, even within specialized centres, and clinical outcomes have been suboptimal.^10^ Moreover, such approaches are not applicable in the setting of infective endocarditis (IE).

In the context of IE, MAC introduces additional diagnostic and therapeutic complexities.^11^ Differentiating thrombi on MAC from vegetations can be challenging. In addition, although surgical management of MAC is inherently complex, the added urgency and septic nature of IE further complicate the procedure. Echocardiographic characteristics, management approaches, and clinical outcomes for patients with IE and MAC remain poorly characterized. Given the limited data on this entity, we retrospectively reviewed all consecutive cases of IE and MAC at our institution, to describe the echocardiographic features of IE on MAC and evaluate associated management strategies and clinical outcomes.

## Methods

### Study design

We reviewed all consecutive patients discussed during the IE multidisciplinary rounds at our institution between July 2021 and July 2024,^12^ and we identified those with either possible or confirmed IE and moderate or severe MAC as assessed by echocardiography. The diagnosis of IE was based on the Duke criteria, using the 2015 version until 2023, and the modified 2023 Duke criteria thereafter.^13^ Patients were excluded if the diagnosis of IE was ultimately refuted by the IE team. Clinical information was obtained from review of patients’ charts. The population was classified into 2 groups based on whether or not the IE involved the mitral valve. The study was approved by our institutional review board, and the requirement for individual patient consent was waived.

### Clinical characteristics

We gathered information on past medical history and comorbidities through review of the electronic medical record, including multidisciplinary rounds notes. Clinical presentation was categorized as heart failure, sepsis, and/or neurologic deficit (defined as the patient being admitted with a transient ischemic attack [TIA], stroke, or altered level of consciousness). These categories were not mutually exclusive. Coronary artery disease was defined as coronary lesions confirmed by a coronary angiogram prior to admission (either treated by percutaneous coronary intervention or coronary artery bypass grafting, or managed medically). Chronic kidney disease was defined as an estimated glomerular function rate below 60 mL/min per 1.73 m^2^. The Charlson Comorbidity Index was calculated for each patient, to quantify comorbidity burden.^14^ In-hospital management and outcome were also extracted from patients’ charts.

### Echocardiography

All transthoracic echocardiography (TTE) and/or transesophageal echocardiography (TEE) was performed using an EPIC ultrasound machine (Philips Healthcare, Andover, MA) and were clinically indicated.^15^ Echocardiographic images were retrospectively reviewed by a level III echocardiographer to confirm the presence of moderate or severe MAC, defined as calcification extending beyond one-third of the posterior mitral annular circumference in the parasternal short-axis view, per our echocardiography laboratory protocol.^5^^,^^6^ Mitral and aortic regurgitation severity was assessed using a multiparametric approach, and MS and aortic stenosis were assessed based on mean pressure gradient.^16^^,^^17^ The presence of an abscess was defined by the identification of nonhomogeneous echogenic or echo lucent perivalvular thickening.^18^ Left ventricular ejection fraction was determined either using the biplane Simpson’s method or visually. Left ventricular ejection fraction was categorized as normal (≥ 60%), mildly reduced (40%-59%), or moderately-severely reduced (< 40%).^15^ Right ventricular ejection fraction was assessed using a multiparametric approach, as recommended.^19^ Systolic pulmonary artery pressure was calculated using continuous-wave Doppler measurement of the tricuspid regurgitation velocity.^20^

### Management and outcomes

Theoretical surgical indications were categorized as follows: refractory heart failure; uncontrolled infection (defined as the presence of an abscess, pseudoaneurysm, fistula, or persistent fever or positive blood cultures despite adequate antibiotic therapy for ≥ 7 days); infection caused by microorganisms at low likelihood of being controlled by antimicrobial therapy alone (including fungi, Methicillin-resistant *Staphylococcus aureus*, vancomycin-resistant *enterococci*, and non-HACEK Gram-negatives in cases of native valve endocarditis; and all *staphylococci* or non-HACEK Gram negatives in cases of prosthetic valve endocarditis); or prevention of embolic events (vegetations ≥ 10 mm with ≥ 1 embolic episodes despite appropriate antibiotic therapy). Surgical decision-making was guided first by the 2015 European Society of Cardiology (ESC) guidelines,^21^ and subsequently by the revised 2023 ESC guidelines.^17^ In-hospital complications were ascertained until the time of discharge; these included embolism, heart failure (requiring intravenous diuresis), acute kidney injury requiring renal replacement therapy, and death.

### Statistical analysis

Continuous variables were expressed as mean ± standard deviation, or median (25%-75% percentile interquartile range [IQR]), as appropriate. Categorical variables were expressed as counts (percentages). Comparisons between groups (mitral IE and nonmitral IE) were performed using the *t*-test, the Wilcoxon rank-sum test, or the χ^2^ test, as appropriate. Mortality rates at 30 days and 1 year between groups were compared using the log-rank test. A 2-tailed *P-*value < 0.05 was considered statistically significant. Statistical analyses were performed using JMP 17 software (SAS Institute, Cary, NC).

## Results

### Study population

**Figure 1:**
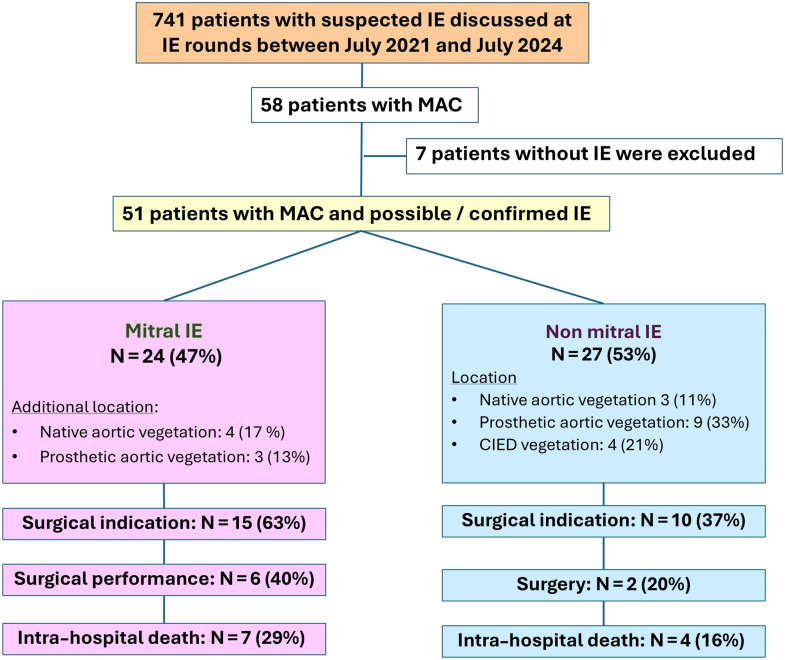
Flowchart of the study population, management and outcome. CIED, cardiac implantable electronic defibrillator; IE, infective endocarditis; MAC, mitral annular calcification.

Of the 741 patients assessed by our IE multidisciplinary team between July 2021 and July 2024, 58 (8%) had moderate or severe MAC. In 7 patients, IE ultimately was ruled out by the IE team. Three patients had no detectable masses on TTE or TEE, and their clinical histories were not suggestive of IE; 2 had imaging findings that were attributed to thrombus on MAC; one patient had a thrombus on a cardiac implantable electronic device (CIED) lead; and the last patient had a noninfective perivalvular leak following a transcatheter aortic valve implantation (TAVI). Ultimately, the study population comprised 51 patients with moderate or severe MAC and either possible or definite IE (Fig. 1).

### Clinical presentation

The mean age was 73 ± 11 years, and 23 patients (45%) were female. By design, none of the patients had a prior history of mitral valve intervention; however, 27 patients (53%) had a prior cardiac intervention. Aortic valve interventions were the most frequent, with 20 patients undergoing a total of 21 procedures: 10 surgical aortic valve replacements (8 with bioprosthetic and 2 with mechanical valves), and 11 TAVI procedures, including one valve-in-valve procedure for a degenerated bioprosthetic valve. Additionally, 11 patients (22%) had a CIED, and 9 patients (18%) had a history of coronary artery bypass grafting.

At admission, 31 patients (61%) presented with sepsis, 16 patients (31%) presented with neurologic symptoms, and 14 patients (27%) presented with heart failure. Blood cultures were positive in 45 patients (88%), with *Staphylococcus aureus* being the most common pathogen (17 patients; 39%), followed by *Streptococcus* species (11 patients; 22%), *Enterococcus* species (11 patients; 22%), and coagulase-negative staphylococci (5 patients; 10%).

IE involved the mitral valve in 24 patients (47%), a native aortic valve in 7 patients (14%), a prosthetic aortic valve in 12 patients (24%), a CIED in 4 patients (11%), and multiple valves in 7 patients (14%; Central Illustration). Eleven patients (22%) had no valvular lesion detectable by echocardiography, and IE was either confirmed through positron emission tomography-computed tomography (PET-CT) or cardiac CT (all in the nonmitral IE group). Compared to the nonmitral IE group, patients with mitral IE were more likely to be female (63% vs 30%, *P* = 0.02) and had a lower prevalence of coronary artery disease (17% vs 63%, *P* < 0.01) and prior aortic valve intervention (17% vs 59%, *P* < 0.01). No difference was present in microbiologic profile overall (*P* = 0.29), with *Staphylococcus aureus* accounting for 38% and 30% of IE cases in the mitral and nonmitral IE groups, respectively (*P* = 0.55), and negative blood culture for 8% vs 15%, respectively (*P* = 0.47; Table 1).

**Central Illustration:**
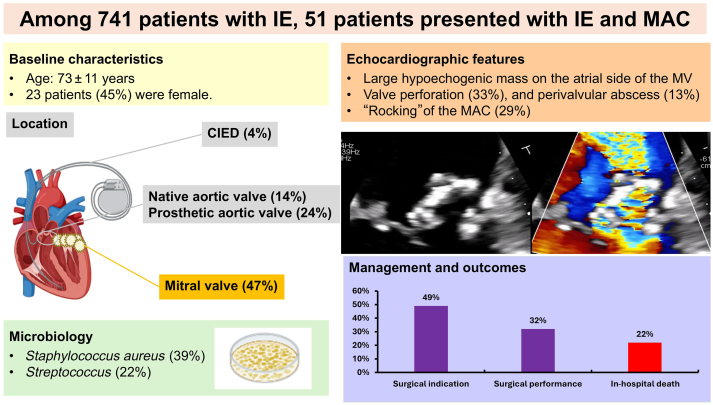
Characteristics and outcomes of the 51 patients with infective endocarditis (IE) in patients with mitral annular calcification. Management and outcomes relate to the entire population. Rate of surgery relates to patients with a surgical indication. CIED, cardiac implantable electronic defibrillator; IE, infective endocarditis; MAC, mitral annular calcification; MV, mitral valve

**Table 1:** Baseline clinical characteristics of the study population

Characteristic	Overall (N = 51)	Mitral IE (n = 24)	Nonmitral IE (n = 27)	*p*
Age, y	73 ± 11	72 ± 12	75 ±10	0.16
Sex, female	23 (45)	15 (63)	8 (30)	0.02
Body mass index, kg/m^2^	30 [22–36]	33 [22–40]	29 [21–34]	0.17
**Cardiovascular risk factors**				
Current tobacco smoking	15 (29)	9 (38)	6 (22)	0.23
Hypercholesterolemia	25 (49)	9 (38)	16 (59)	0.12
Diabetes mellitus	30 (59)	12 (50)	18 (67)	0.23
Hypertension	38 (75)	15 (63)	23 (85)	0.06
**Past medical history**				
Coronary arterial disease	30 (59)	4 (17)	17 (63)	< 0.01
Transient ischemic attack and/or stroke	11 (22)	5 (21)	6 (22)	0.90
Chronic kidney disease	13 (26)	5 (21)	8 (30)	0.47
Hemodialysis	5 (10)	2 (8)	3 (11)	0.74
Cirrhosis	5 (10)	2 (8)	3 (11)	0.73
Chronic obstructive pulmonary disease	6 (12)	4 (17)	2 (7)	0.30
History of cancer	7 (14)	3 (13)	4 (15)	0.81
Cardiac implantable electronic device	11 (22)	3 (13)	8 (30)	0.13
History of atrial fibrillation	25 (49)	10 (42)	15 (56)	0.32
Prior coronary arterial bypass graft	9 (18)	0 (0)	9 (33)	< 0.01
Patient with prior aortic valve intervention	20 (39)	4 (17)	16 (59)	< 0.01
Charlson Comorbidity Index	6 [4–7]	4.5 [4–6.2]	6 [5–7]	0.07
**Laboratory results**				
Creatinine at admission, μmol/L	101 [69–202]	102 [60–150]	90 [76–279]	0.25
Albumin, g/dL	30 ± 5	29 ± 5	31 ± 5	0.38
White blood count, G/L	11 [9–13]	11 [9–14]	11 [8–13]	0.73
**Microorganism**				0.29
*Staphylococcus aureus*	17 (39)	9 (38)	8 (30)	
*Streptococcus*	11 (22)	7 (29)	4 (15)	
*Enterococcus*	11 (22)	5 (21)	6 (22)	
Coagulase negative *Staphylococcus*	5 (10)	2 (8)	3 (11)	
Other	7 (14)	1 (4)	6 (22)	
Negative blood culture	6 (12)	2 (8)	4 (15)	0.47
**Clinical presentation**				
Heart failure	14 (27)	7 (29)	7 (26)	0.80
Sepsis	31 (61)	15 (63)	16 (59)	0.81
Neurologic deficit	16 (31)	9 (38)	7 (26)	0.37

Values are number of patients (percentage), mean ± standard deviation, or median [interquartile range], unless otherwise indicated.

IE, infective endocarditis.

### Echocardiographic features

**Figure 2:**
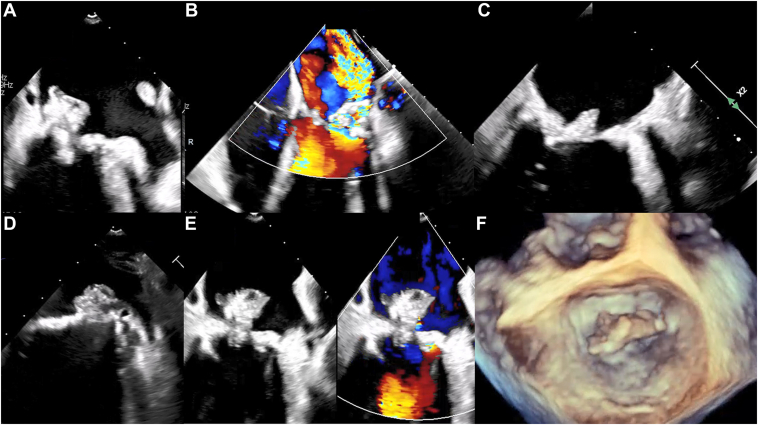
**Example of infective endocarditis in patients with mitral annular calcification (MAC) using transesophageal echocardiography.** (**A**) Large heterogeneous vegetation on the medial side of the mitral annulus. (**B**) Valvular perforation, with severe mitral regurgitation on the lateral side of the mitral annulus. (**C**) Heterogeneous hypodense vegetation on MAC. (**D**) Succulent heterogeneous vegetation on MAC. (**E**) Color comparison of the mitral valve showing a large heterogeneous vegetation overlying the calcified annulus. (**F**) Large vegetation visualized on 3-dimensional transesophageal echocardiography.

TTE was performed in all cases, and TEE was performed in all but 10 patients. Key echocardiographic features are summarized in Table 2. Among the 24 patients with mitral IE, 17 (71%) had isolated mitral valve involvement, and 7 (29%) had concomitant aortic valve infection, affecting a native valve in 4 cases and a prosthetic valve in 3 cases. Vegetations were located on the mitral leaflets in 13 patients (54%), on the mitral annulus in 8 patients (33%), and on both the mitral leaflets and the annulus in 3 patients (13%). Additionally, consistent with the frequent extension of calcification, vegetations were located on the posterior leaflet in 17 patients (71%), the anterior leaflet in 5 patients (21%), and the subvalvular apparatus in 2 patients (8%). Vegetations were isolated in 15 patients (63%) and were multiples in 8 patients (33%). Vegetation size was significantly larger than in the counterpart nonmitral IE group (13 vs 8 mm, *P* = 0.02). Highly mobile vegetations prolapsing into the left ventricle were noted in 10 patients (42%). A mitral valve perforation was reported in 8 patients (33%), and a perivalvular abscess was noted in 3 patients (13%). Moderate or greater MR was present in 13 patients (54%). A noteworthy point is that a succulent aspect of the vegetation mass with abnormal MAC mobility, described as "rocking," was observed in 7 patients (29%; Fig. 2; Videos 1-3 , view videos online).

**Table 2:** Echocardiographic findings

Finding	Overall (N = 51)	Mitral IE (n = 24)	Nonmitral IE (n = 27)	*p*
Left ventricular ejection fraction, %				
≥ 60	37 (73)	20 (83)	17 (63)	0.03
40–59	9 (18)	4 (17)	5 (19)	
< 40	5 (10)	0 (0)	5 (19)	
Systolic pulmonary artery pressure, mm Hg	41 ± 13	43 ± 12	39 ± 15	0.41
Vegetation size, mm	11 [7–16]	13 [11–18]	8 [4–14]	0.02
Vegetation size ≥ 10 mm	17 (33)	14 (58)	3 (11)	0.01
Mitral regurgitation ≥ moderate	15 (29)	13 (54)	2 (7)	< 0.01
Transmitral mean gradient	4 ± 3	5 ± 2	4 ± 3	0.11
“Rocking” of the mass	7 (14)	7 (29)	—	
Perivalvular mitral abscess	3 (6)	3 (13)	—	
Aortic regurgitation ≥ moderate	4 (8)	3 (13)	1 (4)	0.24
Periaortic abscess	8 (16)	3 (13)	5 (19)	0.55
Aortic mean gradient, mm Hg	9 [5–28]	5.5 [4–15]	19 [7–29]	0.04
Tricuspid valve lesion	2 (4)	1 (4)	1 (4)	0.93
Tricuspid regurgitation ≥ moderate	3 (6)	1 (4)	2 (7)	0.67

Values are number of patients (percentage), mean ± standard deviation, or median [interquartile range], unless otherwise indicated.

IE, infective endocarditis.

### Management

Among the 24 patients with mitral IE, 15 (63%) had at least one theoretical surgical indication. Surgery ultimately was performed in 6 patients, representing 24% of the overall mitral IE population and 40% of those with a surgical indication. The most common indications for surgery were prevention of embolic complication, in 9 patients (60%), and refractory heart failure, in 8 patients (53%). Of note, anticoagulation therapy was recommended in 5 patients (21%). Reasons for surgical turndown included technical challenges as well as comorbidities, as illustrated by their older age (median 77 years, IQR [75-85] vs 64 years, IQR [57-66], *P* = 0.02) and higher Charlson Comorbidity Index, vs the 6 patients who were operated on (median 6.0, IQR [4.0-7.0] vs 4.0, IQR [3.25-4.0], *P* = 0.04). Compared with patients with nonmitral IE, those with mitral IE more often had a theoretical indication for surgery (63% vs 37%, *P* = 0.07), driven largely by a higher rate of surgery for embolism prevention (60% vs 10%, *P* < 0.01). However, surgical performance rates were not significantly different although they were numerically higher in mitral IE patients (40% vs 20%, *P* = 0.29). The management and outcomes of both groups are detailed in Table 3.

**Table 3:** Management and outcomes

	Overall (N = 51)	Mitral IE (n = 24)	Nonmitral IE (n = 27)	*p*
**Endocarditis team decision**				
Theoretical surgical indication	25 (49)	15 (63)	10 (37)	0.07
Surgery performed	8 (32)	6 (40)	2 (20)	0.44
**Indication for surgery**				
Refractory heart failure	12 (48)	8 (53)	4 (40)	0.51
Locally uncontrolled infection	5 (20)	2 (13)	3 (30)	0.31
Microorganism	6 (24)	2 (13)	4 (40)	0.13
Prevention of embolism	10 (40)	9 (60)	1 (10)	< 0.01
**In-hospital outcomes**				
In hospital mortality	11 (22)	7 (29)	4 (15)	0.32
In-hospital stroke and transient ischemic attack	5 (10)	3 (13)	2 (7)	0.48
All stroke or transient ischemic attack∗	21 (41)	12 (50)	9 (33)	0.23
Spleen embolism	6 (12)	5 (21)	1 (4)	0.05
Congestive heart failure	17 (33)	11 (46)	6 (22)	0.07

Values are number of patients (percentage), unless otherwise indicated.

∗ Including prehospital and in-hospital stroke.

### Complications and outcomes

**Figure 3:**
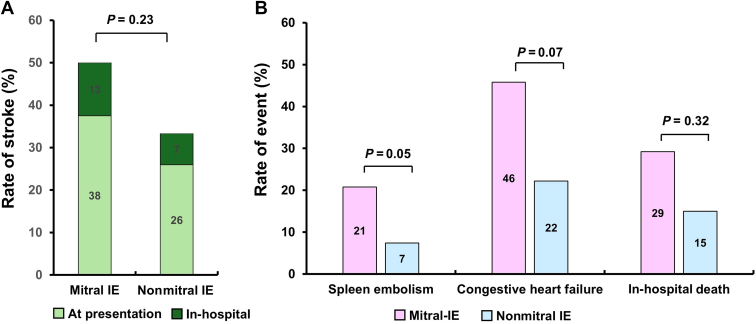
In-hospital complications. (**A**) Rate of stroke and transient ischemic attack before and during admission. (**B**) Rate of splenic embolism, congestive heart failure, and in-hospital mortality. IE, infective endocarditis.

Of the 24 mitral IE patients, 9 (38%) presented with either stroke or TIA at admission, and an additional 3 patients (13%) experienced in-hospital neurologic embolism while admitted (Fig. 3). Eleven patients (46%) developed congestive heart failure. In-hospital death occurred in 7 patients (29%) of the mitral-IE cohort. Stroke and/or TIA events, including those at presentation, were numerically more frequent in the mitral IE group than in the nonmitral IE group (50% vs 33%, respectively, *P* = 0.23). Eleven patients (46%) of the mitral IE group presented with congestive heart failure, compared to 6 patients (22%) in the nonmitral IE group (*P* = 0.07). In-hospital mortality was not statistically different between the mitral and nonmitral IE groups (29% vs 15%, respectively, *P* = 0.32). Similarly, mortality rates at 30 days and 1 year were not different between the 2 groups (33% vs 23% and 50% vs 40%, respectively (log-rank *P* = 0.47). Complications and in-hospital outcome details are presented in Table 3.

## Discussion

The main findings of this study can be summarized as follows: (i) patients with MAC represent approximately 1 in 10 IE cases at our institution, with about half of these involving the mitral valve; (ii) MAC-associated IE often presents on echocardiography as large, mobile masses attached to the MAC and/or posterior leaflet, with a distinct appearance, and is frequently associated with leaflet perforation or abscess; (iii) in patients with IE and MAC, stroke and/or TIA and congestive heart failure were common complications; and (iv) although a theoretical surgical indication was present in two-thirds of patients, surgery was performed in only 40% of them.

### Rate of MAC in patients with IE

This study is among the first to investigate the incidence, clinical presentation, and management of IE in patients with MAC. In our study, of 741 patients with suspected IE discussed at IE rounds, 7% presented with MAC and possible or confirmed IE. This result highlights the common presence of MAC in patients with IE, as in the general population. Previous results reported a prevalence of MAC of 8%-15% in the general population, and of up to 40% or higher in elderly individuals.^22^ Patients with MAC represent a high-risk population, characterized by advanced age and significant comorbidities.^4^ In our cohort, the mean age was 73 years; 49% of patients had hypercholesterolemia, 59% had diabetes, and 75% had hypertension. Coronary artery disease was present in 59%, a history of stroke or TIA in 22%, and chronic kidney disease in 26%.

### Echocardiographic findings and challenges

Approximately half of the patients with IE and MAC presented with mitral valve IE, whereas the other half had IE involving other structures, such as the aortic valve, a prosthetic aortic valve, or a CIED. A point that remains unclear from our study is whether individuals who developed IE on MAC had a preexisting thrombus that became secondarily infected, or if the MAC itself serves as an anatomic substrate and/or nidus predisposing the patient to IE. Of note, AS and MAC are frequently associated, and the lower prevalence of prior valve replacement in patients with mitral valve IE likely reflects the greater susceptibility of prosthetic material to infection, compared with native MAC tissue. IE associated with MAC often presented with large, mobile vegetations and severe valvular and perivalvular complications. Moderate or severe MR was observed in 54% of patients, and 29% exhibited a “rocking” motion of the annulus, suggesting abscess formation and/or perforation. Abnormal motion of valvular prostheses, particularly in the context of IE, has been well described as a "rocking” motion and is typically indicative of structural disruption or peri-annular complications.^23^ When observed in patients with MAC, this finding also should raise suspicion for an abscess or peri-annular collection, and in our experience, requires prompt consideration for surgery. Differentiating IE-related vegetations from thrombi can be challenging, leading some patients to receive anticoagulation alongside antibiotics, especially when IE was only possible.^24^ However, anticoagulation in IE carries risks, such as hemorrhagic transformation of embolic strokes, rupture of cerebral or visceral mycotic aneurysms, and excessive perioperative bleeding in patients undergoing urgent valve surgery, and reliable echocardiographic markers to distinguish vegetations from thrombi on MAC remain elusive. In our experience, TEE was essential for diagnosis, as dense calcifications caused acoustic shadowing that limited TTE diagnostic value. TEE should therefore be considered systematically in patients with MAC when IE is suspected, to prevent missed diagnoses that may delay the initiation of antibiotic therapy and lead to worse outcomes. Multimodality imaging, such as PET-CT or cardiac CT, can help by supporting diagnosis of IE.^25^

### High risk of embolic events and mortality

Patients with mitral valve IE experienced a high rate of embolic events, with stroke occurring in nearly 50% of cases—significantly higher than the 5.3% stroke rate reported in the ESC EURObservational Research Programme (EORP) cohort of 3116 IE patients.^26^ The large size of mitral IE vegetations (> 10 mm in 61% of cases, and > 15 mm in 26% of cases) is a key risk factor for embolism. In a multicentre study of 2171 patients with left-sided IE, vegetation size was reported to be > 10 mm in 42% of cases.^26^ The in-hospital mortality rate tended to be higher in the mitral IE group (29%) compared to that in the nonmitral IE group (16%), although this difference did not reach the level of statistical significance. For context, the ESC EORP cohort reported an overall in-hospital mortality rate of 17%, which is more closely aligned with that of the nonmitral IE group in our study.^27^

### Surgery

Although 63% of patients with mitral IE had theoretical surgical indications, surgery was ultimately performed in only 40%. In comparison, data from the ESC EORP cohort showed that 51% of patients with IE underwent surgery overall, and among those with a surgical indication, 74% received an intervention.^27^ This disparity likely reflects the high surgical risk in patients with MAC, many of whom were deemed unsuitable for intervention due to both the technical challenges of managing MAC with IE and these patients’ heavy comorbidity burden, as reflected by the older age and higher Charlson Comorbity Index in patients turned down for surgery. Indeed, the presence of MAC may prompt a preference for medical management, given the significantly elevated risk of perioperative mortality, stroke, and acute kidney injury, factors that likely contribute to the poorer outcomes observed in this population.^10^ Specific surgical interventions (decalcification and reconstruction of mitral valve annulus, intra-atrial prosthesis placement) have been proposed to tackle the risk of mitral valve surgery in patients with MAC. However, the rate of patients who are denied surgery in this context remains important.^28^ Several studies have shown that the decision to not intervene when intervention was indicated was associated with a 3-fold increased mortality rate.^27^^,^^29^

### Limitations

Several limitations must be acknowledged. First, this study was retrospective and was conducted at a single centre, although thorough individual chart reviews ensured comprehensive and accurate data collection. Second, the observational design limits the ability to draw definitive causal inferences relating treatment strategies to outcomes, including mortality. The identification of guideline-supported surgical indications was based on chart review and followed a theoretical framework that does not necessarily reflect the more nuanced clinical judgment that considers the overall risk-benefit ratio in surgical decision-making. For example, in patients with IE following TAVI, or in elderly patients, medical management is often preferred in clinical practice, even when theoretical indications for surgery are present. Third, comparison of mitral valve IE in patients with vs without moderate or severe MAC would have been informative, but this could not be done easily in the absence of a structured database. Fourth, MAC severity was assessed semiquantitatively using echocardiography. Cardiac CT can provide quantitative information on both the circumferential and myocardial extent of MAC, but it was rarely performed in our patients; its role warrants further investigation. Finally, the relatively small sample size may have limited the statistical power to detect significant differences between groups.

### Clinical implications

Evaluation and management of patients with IE on MAC are complex and remain suboptimal. Diagnosis relies on a combination of clinical history and echocardiographic findings, with TEE recommended systematically in this setting and repeated to monitor evolutional changes. The presence of large vegetations, a “succulent” aspect of mass on MAC or a “rocking” aspect of the MAC, highly suggestive of annular complications, should prompt consideration of surgery if the patient is an appropriate candidate. However, poor outcomes in this subset result from multiple factors, including advanced age, high comorbidity burden, elevated stroke risk, surgical technical challenges, and reluctance to undertake high-risk procedures. Future strategies should emphasize earlier diagnosis, refined imaging modalities including cardiac CT and PET, and tailored surgical approaches to improve outcomes. The role of transcatheter therapies appears to be limited, at least in the acute setting.

## Conclusion

This study highlights the substantial burden of MAC among patients with IE, with approximately half of the cases involving the mitral valve. Mitral IE was frequently associated with large, mobile vegetations, valve perforation, or annular abscesses, and a heterogeneous, lobulated (“succulent”) appearance of the masses and a “rocking” motion, features linked to a high rate of embolic events. Although surgical indications were often present, surgery was performed in only a minority of patients, likely due to the elevated surgical risk inherent to this population and the surgical risk intrinsically associated with mitral valve surgery in patients with MAC. These findings underscore the need to optimize management strategies in this complex and high-risk group.

## Supplementary Material

Video 1 Large mass on the atrial side of the valve, associated with an excessive mobility (rocking) and perforation of the posterior aspect of the mitral annulus

Video 2 Rocking motion of the MAC with heterogeneous and mobile small masses on the MAC.

Video 3 Large vegetation on the atrial side of the medial mitral annulus. The mass sitting on the MAC is heterogeneous with a “succulent” aspect.
